# Refractory multisystemic sarcoidosis, a diagnosis and treatment challenge: a case report

**DOI:** 10.1186/s13256-023-03996-w

**Published:** 2023-06-29

**Authors:** Jorge Luis Rodas Flores, Enrique Peral Gutiérrez de Ceballos, Blanca Hernández-Cruz, Alejandro Hernán Alvarez Muñoz, Jesús Machuca-Aguado, Salvador Recio Gallardo, José Javier Perez Venegas

**Affiliations:** 1 Rheumatology Department, Virgen Macarena University Hospital, Av Dr. Fedriani 3, 41009 Seville, Spain; 2 Internal Medicine Department, Virgen Macarena University Hospital, Av Dr. Fedriani 3, 41009 Seville, Spain; 3 Pathological Anatomy Department, Virgen Macarena University Hospital, Av Dr. Fedriani 3, 41009 Seville, Spain; 4 Radiology Department, Virgen Macarena University Hospital, Av Dr. Fedriani 3, 41009 Seville, Spain

**Keywords:** Refractory sarcoidosis, Methotrexate, Adalimumab, Infliximab, Case report

## Abstract

**Background:**

Sarcoidosis is a multisystemic granulomatous disease of unknown origin. It is characterized by abnormal activation of lymphocytes and macrophages with the formation of granulomas. Most cases have asymptomatic pulmonary involvement. In case of symptoms, they have an excellent response to glucocorticoid therapy. We present a case of sarcoidosis with multi-organ involvement, refractory to multiple treatments including biological. Partial remission was achieved in it.

**Case presentation:**

We report an interesting case of a 38-years-old Spanish woman treated by Heerfordt’s syndrome (uveitis, parotiditis, fever and facial palsy) plus pulmonary hiliar adenopathy. A sarcoidosis diagnosis was confirmed by lung biopsy. She was initially treated with an 8 weeks course of medium dose oral glucocorticoids and tapered over 8 weeks with improvement. After the suspension of glucocorticoids a relapse occurs with severe ocular involvement and suspicion of neurological involvement. The patient received multiple lines of treatment with poor response. Finally, after the combination of cyclophosphamide with infliximab, the uveitis resolved, improving the neurological symptoms.

**Conclusions:**

Sarcoidosis is a benign disease in most cases. In a small percentage of cases behaves aggressively, requiring early diagnosis and immunosuppressive treatment to avoid sequelae. An adequate immunosuppressive therapy based on Anti TNF drugs should be started to minimize damage and improve the quality of life.The choice of treatment depends on the type and severity of the disease.

## Background

Sarcoidosis is a multisystemic granulomatous disease of unknown etiology [1]. It is characterized by the abnormal activation of CD4 T lymphocytes and macrophages. These cells accumulate in the affected tissues, where they lead to the formation of granulomas. These cells produce various proinflammatory cytokines responsible for the clinical manifestations and complications of the disease. Sarcoidosis can affect several organs and systems, and the lungs are the most frequently affected [2, 3].

Sarcoidosis is only treated in a subgroup of patients to prevent damage to the affected organ. Treatment prevents long-term complications and improves the quality of life [4]. Currently, there are several therapeutic options that must be individualized according to the patient. They can range from surveillance in asymptomatic cases, to intensive immunosuppressive treatment [4, 5]. We report a clinical case of sarcoidosis with different levels of involvement and refractory to conventional treatment.

## Case presentation

**Fig. 1:**
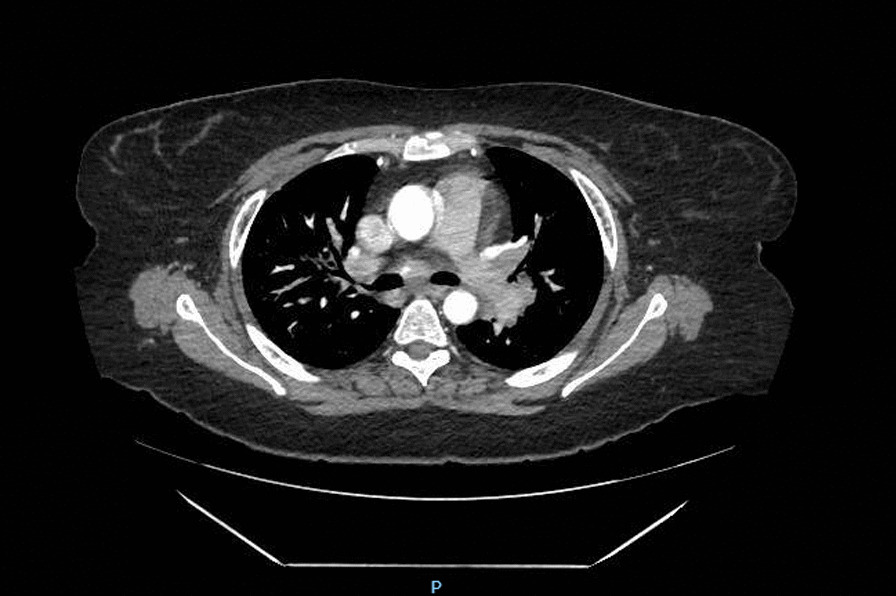
Bilateral hilar adenopathies present in chest CT

A 38-year-old Spanish woman, current smoker, with hypothyroidism and obesity (90 kg, BMI 35) was referred in March 2021 from Primary Care to the Internal Medicine Department. She had persistent fever for 5 days (38°–39 °C) and asthenia. The first evaluation revealed the presence of fever, parotid enlargement, bilateral anterior uveitis, and peripheral facial palsy on the left side. In the chest X-ray, it was noticed the presence of bilateral hilar adenopathies, with better visualization in the thorax tomography (Fig. 1). The blood tests showed an angiotensin converting enzyme (ACE) value of 77 U/L (normal value 12–40); the rest of parameters: hemogram, liver and renal test, erythrocyte sedimentation rate (ESR), c reactive protein (CRP), serum calcium, ferritin, proteinogram, serology for human immunodeficiency virus (HIV), hepatitis B virus (HBV), hepatitis C virus (HCV), hepatitis E virus (HEV), QuantiFERON-TB Gold Test, antinuclear antibodies and several autoimmune tests were negative. Urine, blood and sputum cultures were negative.

With the clinical manifestations and the complementary tests, the diagnosis of Heerfordt syndrome as manifestation of acute sarcoidosis was established. Since the syndrome with pulmonary hiliar adenopathies is considered pathognomonic of sarcoidosis, it was decided to treat without histopathology confirmation. Oral prednisone was prescribed at a dose of 30 mg/day, for two months with subsequent control.

In the first evaluation after a month of treatment, she was better. The fever and the parotid enlargement had subsided, the left facial palsy improved. The ophthalmological evaluation showed resolution of the uveitis, with normal ocular examination. However, she had new clinical data: headache, dizziness, and upper limbs paresthesia. Her blood count, chemistry tests, ESR, CRP, urine and thorax X-Ray were normal. A brain magnetic resonance imaging (MRI) showed normal results. In addition, the upper limb electroneurogram showed sensitive involvement in the median nerve at the level of the right carpal tunnel. As no neurological involvement of sarcoidosis was demonstrated and other possible causes of the symptoms were ruled out, prednisone was continued at 30 mg/day for a month more with subsequent taper until discontinuation in 2 months.

In June 2021 she was evaluated, and all of her symptoms had resolved. However, four days after discontinuing glucocorticoids, she consulted again for decreased vision, pain and ocular erythema. A bilateral panuveitis was diagnosed (positive Tyndall, posterior synechiae, vitreous condensation and papilla thickening) by eye fundus examination and Optical Coherence Tomography. She was treated with topical glucocorticoids and cycloplegics. One week later, the patient presented worsening of her uveitis symptoms in addition to fever of 38 °C. The patient was admitted and treated with intravenous methylprednisolone pulses at a dose of 500 mg/day for 3 days. The ophthalmologic response was very satisfactory, with remission of pain, and almost complete recovery of visual acuity. After discussing the case in the Multidisciplinary Committee of Autoimmune Diseases, it was decided to add to the treatment methotrexate (20 mg/week SC) and adalimumab (40 mg/2 weeks SC), in addition to prednisone (20 mg/day PO).

A month later the patient reported improvement of her symptoms: no fever, improvement of her visual acuity. Her paresthesias had also improved. It was decided to maintain the dosage of the prescribed medication.

In August 2021 she consulted several times due to persistent headache, fever, and asthenia. It was decided to increase the daily dose of oral prednisone from 20 to 1 mg/Kg/day (80 mg) for 10 days in addition to methotrexate and adalimumab. A month later she continued with the same symptoms and was admitted for monitoring and to rule out other possible etiology (i. e. central nervous system infection). During the admission the patient presented with a fever of 38 C and persistence of holocranial headache. Occasionally she presented anomia and paraphasias. Ophthalmological examination showed new signs of uveitis. The patient reported adequate compliance with methotrexate and adalimumab treatment. Her blood tests showed hemoglobin 9.8 g/dL (normal value 13–16), ESR 46 mm/h (normal value 0–20), CRP 0.7 mg/L (normal value < 5) aspartate transaminase (AST) 140 IU/L (normal value 0–37), alanine transaminase (ALT) 225 IU/L (normal value 0–40), angiotensin converting enzyme (ACE) 20 U/L (normal value 12–40) Ca 3.9 mEq/L (normal value 8.5–10.5). Urine, blood and sputum cultures were negative. Other tests: ferritin, proteinogram, serology for HIV, HBV, HCV, HEV, QuantiFERON-TB Gold Test, antinuclear antibodies and autoimmune tests were normal or negative. Lumbar puncture was performed with 30 leukocytes (90% mononuclear), glucose, proteins, and normal adenosine deaminase. In addition to negativity for Gram, acid and alcohol fast bacilli (AAFB), polymerase chain reaction (PCR) for enterovirus and mycobacteria, herpes simplex virus (HSV), varicela herpes virus (VHZ); antineuronal antibodies were negative.

**Fig. 2:**
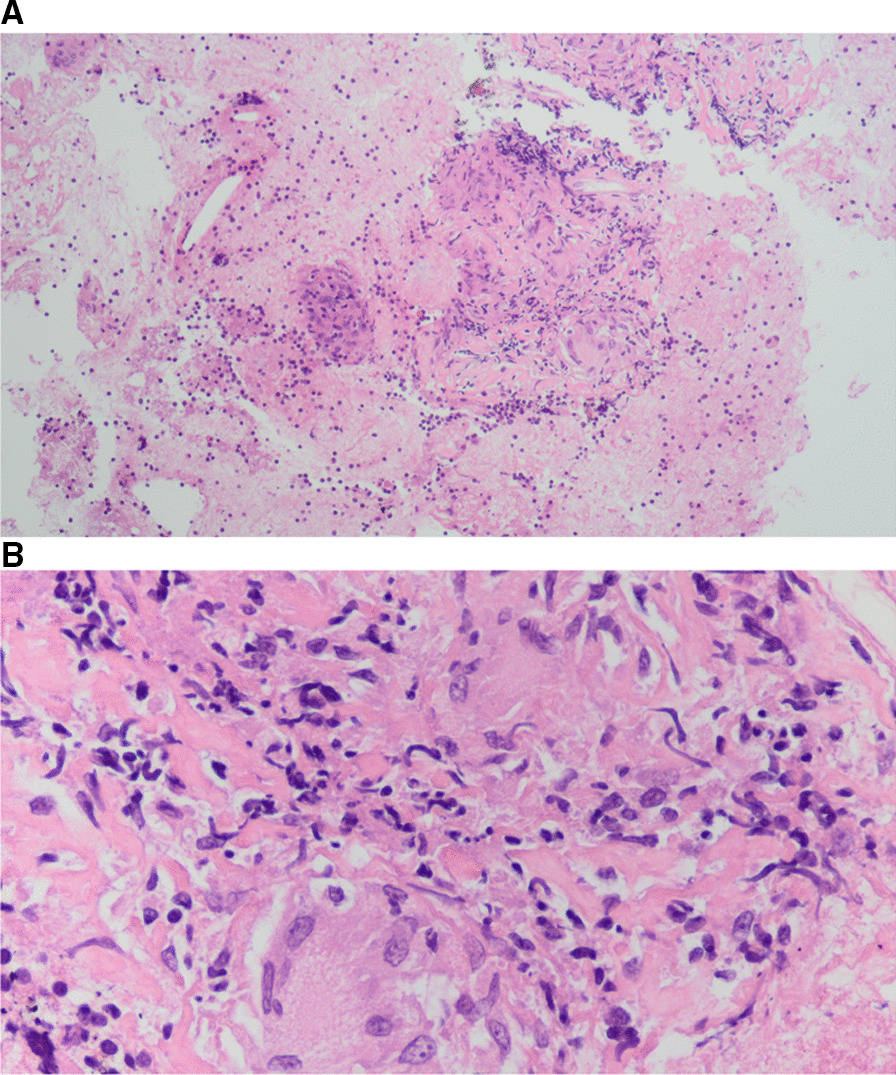
Sample of subcarinal adenopathy, showing sarcoid-type granulomatous lymphadenitis (**A**, **B**)

The electroencephalogram was normal. Brain MRI and AngioMRI showed scarce punctate images with signal of hyperintensity in subcortical white matter of frontal predominance in relation to possible demyelination/nonspecific gliosis, without foci of restriction, space-occupying lesions, or hemorrhagic foci. Echobronchoscopy showed subcarinal lymphadenopathies. A needle aspiration biopsy of hiliar thoracic lymphadenopathies was performed, and the result showed granulomatous lymphadenitis of sarcoid type (Fig. 2). Cytology and microbiology samples were negative. Flow cytometry showed no alteration of the T lymphocytes, non-Hodgkin B lymphoma was ruled out.

**Fig. 3:**
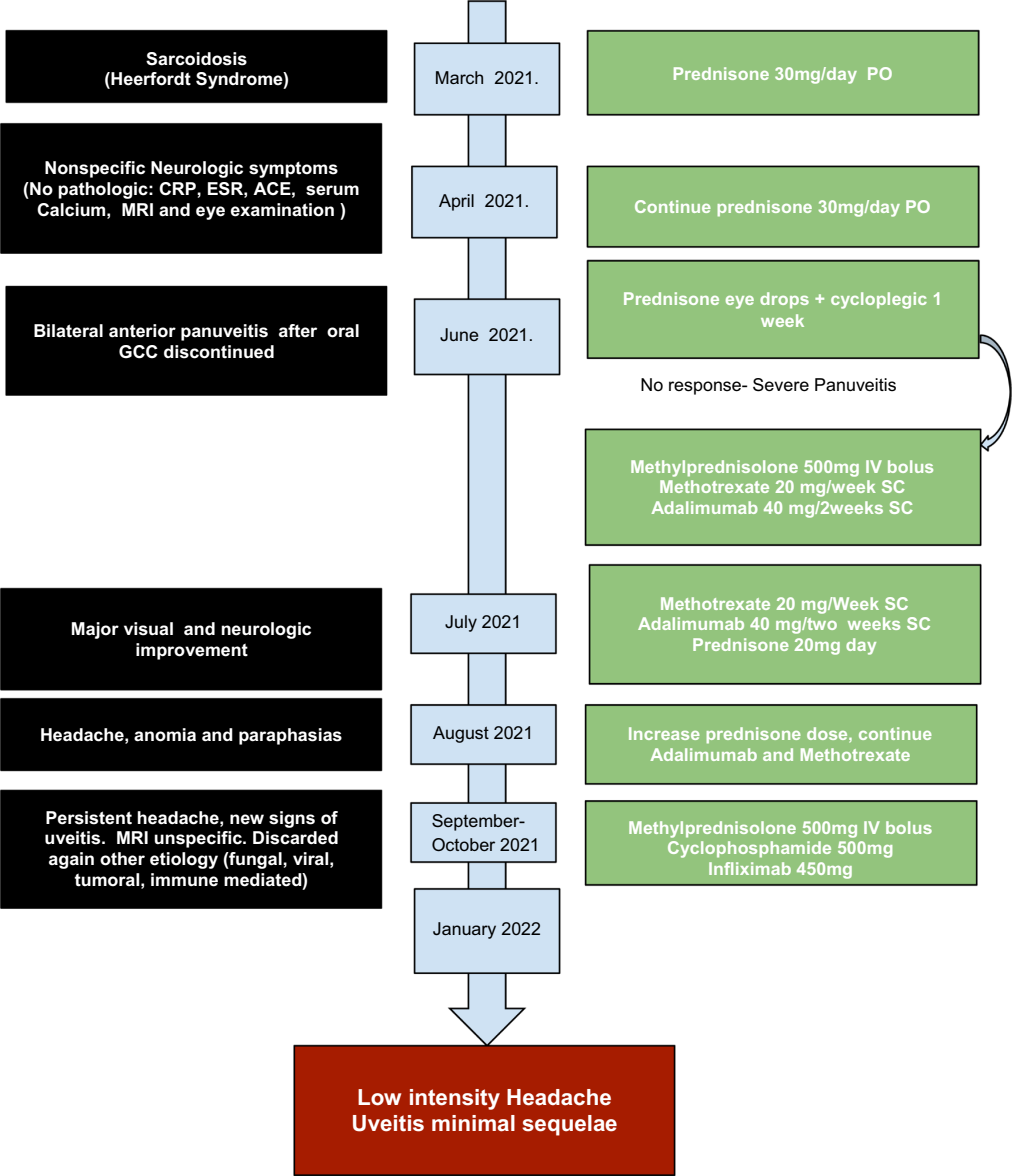
Timeline of the disease process. *GCO* oral glucocorticoids, *MRI* magnetic resonance imaging, *CRP* C reactive protein, *ESR* erythrocyte sedimentation rate, *ACE* angiotensin converting enzyme

The case was discussed again in the Multidisciplinary Committee of Systemic Autoimmune Diseases. It was decided to discontinue methotrexate, due to hepatotoxicity. Given the suspicion of neurological sarcoidosis, adalimumab was changed to cyclophosphamide plus infliximab. After obtaining informed consent, methylprednisolone boluses were prescribed (500 mg/day for 3 days IV), followed by cyclophosphamide (500 mg/ each 2 weeks IV) and infliximab (450 mg/each cycle IV—5 mg/Kg weight—at weeks 0, 2, 6 and then every 8 weeks. Subsequent dose of prednisone of 15 mg/day PO. After 3 months with this treatment, she had no fever normal, had normal visual acuity, and the headache decreased. As a sequel her right eye had mydriasis and snowballs in the eye fundus examination. The patient noticed an improvement in the intensity of the initial symptoms of her disease with the treatments administered. However, she noticed a decrease in her quality of life. The patient follow-up continues. Timeline of the disease process is described in Fig. 3

## Discussion

The diagnosis of sarcoidosis is made when a clinical, radiological, and histological profile is fulfilled. It is confirmed with the demonstration of non-caseating granulomas in the anatomopathological study, and when another pathology has been ruled out [6]. Histology may be dispensable for diagnosis in cases of lupus pernio or acute sarcoidosis, either as Lofgren syndrome (fever, erythema nodosum, hilar adenopathies and arthritis) or Heerfordt syndrome (uveitis, parotiditis, fever and facial palsy), as occurred in the case described [7, 8].

The decision to treat or not the patient should be individualized, according to the clinical profile. Treatment should be based on the type and severity of the involvement. It is important to establish the affected organs, since the efficacy of the treatment may be different. In those cases with severe pulmonary involvement (decrease in respiratory function tests, worsening in Chest Computed Tomography or chest X-Ray, pulmonary hypertension), cardiac (high-grade heart block, heart failure), neurological (central nervous system and peripheral, aseptic meningitis), ophthalmological (uveitis), endocrinological (diabetes insipidus), hepatic, renal, and hypercalcemia; early and effective treatment should be started to prevent damage [2, 6, 7].

In our case, the patient has severe ophthalmological involvement, fever, pulmonary symptoms, and probable neurosarcoidosis, we have followed the clinical practice guidelines for treatment of the European Respiratory Society 2021. The first line of treatment is the use of oral medium dose of glucocorticoid for 8 weeks, until an adequate response is achieved [8] with subsequent taper. In those cases that are resistant or require a corticosteroid sparing effect due to the adverse effects, conventional immunosuppressant should be added as a second line of treatment, either methotrexate, azathioprine, or mycophenolate mofetil [9].

There is no established definition for refractory sarcoidosis in the literature. Some authors consider it when there is no response to glucocorticoids and conventional immunosuppressants, as in the present case [10].

The use of biologics is reserved for those cases in which glucocorticoids and immunosuppressants have not been effective in controlling the disease. In this case the anti-TNF drugs are the best as a third line of treatment. If the involvement is pulmonary, cutaneous, cardiac, or nervous system, the biologic of choice is infliximab or its biosimilar, due to its greater efficacy and low toxicity profile. adalimumab is reserved as a second option of biologic treatment for cases of failure to infliximab [4, 9].

If the patient does not respond to the anti-TNF drugs, a fourth line of drugs can be considered, including rituximab, apremilast, tocilizumab or Janus kinase inhibitors. Although all of them with low evidence of efficacy and awaiting the results of further clinical trials [10].

In cases of ocular sarcoidosis, its initial management is similar to that described for other locations. The first line is the use of glucocorticoids in a descending regimen. As a second line immunosuppressive drugs such as methotrexate preferably, leflunomide or azathioprine can be used. Cyclosporine and tacrolimus are other options to consider [11, 12]. Regarding biologic therapy, adalimumab was the first anti-TNF approved by the FDA for the management of non-infectious uveitis in 2016. It has been the first option of treatment to be used in refractory ocular sarcoidosis in recent years. However, the use of infliximab in several trials as the third line has shown similar effectiveness [12–15]. In our patient, it was decided to prescribe adalimumab as the first option for the severe panuveitis, with an adequate visual response. Our Multidisciplinary Committee of Systemic Autoimmune Diseases manages a uveitis clinic with extensive and good experience with adalimumab. As an alternative therapy for the management of non-infectious uveitis, the use of Anti IL6 (tocilizumab or sarilumab), anti CD20 (rituximab) or Anti IL17 (secukinumab) has been tested in small case series, and a favorable response has been observed, although clinical trials are still pending [16–18].

When a patient with sarcoidosis fails due to ineffectiveness of an anti-tnf, it is important to consider the lack of immunogenicity. This is a common practice in patients with inflammatory bowel disease [10, 19]. The trough levels of the biological in the blood are measured and the presence of antibodies is determined at the same time. If low levels of the drug and high levels of antibodies against the drug are detected, it is immunogenicity. In this case, it is recommended to double the dose of anti-tnf. On the other hand, if high levels of the drug are detected, the recommendation is a change of therapeutic target. In obese patients, as in our case, it is common to find low levels of anti-TNF [19, 20].

The initial clinical and radiological finding in our case, led us to an early diagnosis of sarcoidosis. However, the subsequent atypical evolution, the refractoriness of her symptoms, and the partial response to the administered therapy represented a great limitation in its management, despite having discarded the possibility of a different etiology.

As a summary, a table has been prepared to outline the different levels of treatment according to localization, response, and patient characteristics (Table 1).

**Table 1:** Suggestion of the different levels of treatment to consider in the management of sarcoidosis according to location, response, and patient characteristics [8, 9, 11]

Line of treatment	Treatment	Commnet
First line	• Prednisone/prednisolone 20 mg qd, then 5–10 mg qd	Start at the lowest dose possible, monitor bone density, blood glucose, blood pressure
Second line	• Methotrexate 10–15 mg once weekly • Leflunomide 10–20 mg qd • Azathioprine 50–250 mg qd • Mophetil micofenolate 500–1500 bid • Hidroxychloroquine 200–400 mg qd	Use methotrexate preferably; for methotrexate and leflunomide monitor renal and hepatic function and cytopenias. Avoid mycophenolate mofetil as much as possible due to lower results. Ophthalmological surveillance with hydroxychloroquine
Third line	• Infliximab 3–5 mg/Kg dose at 0, 2, 6 week; then q8 weeks • Adalimumab 40 mg q2 weeks • Rituximab 500–1000 mg q6 months	Use infliximab preferably. Adalimumab preference for ocular sarcoidosis. Avoid anti-TNF in heart failure, active tuberculosis, demyelinating neurological disease, history of neoplasia. Perform viral hepatitis test and IgG control for Rituximab

## Conclusions

We reported a case of a sarcoidosis with pulmonary, ocular, and probable nervous system involvement, with poor response to conventional treatment. In whom more than one biologic therapy was used until an acceptable response was achieved. Most cases of sarcoidosis are mild, and its treatment does not entail a major problem for the physician. On other occasions, the involvement can be multisystemic and severe. In this situation it is important to make a correct diagnosis and early treatment directed according to the affected organ, the patient must have a close monitoring to avoid complications and sequelae leading to decreased quality.

## Data Availability

Data reported on in this study are not publicly available as they were directly obtained from the patient’s electronic medical record. All publicly available cited works can be found in the References section.
